# Design of Reservoirs Enabling Stress-Induced Sequential Release Systems

**DOI:** 10.3390/pharmaceutics14122611

**Published:** 2022-11-26

**Authors:** Osamah Altabal, Christian Wischke, Andreas Lendlein

**Affiliations:** 1 Institute of Active Polymers and Berlin-Brandenburg Center for Regenerative Therapies, Helmholtz-Zentrum Hereon, Kantstr. 55, 14513 Teltow, Germany; 2 Institute of Chemistry, University of Potsdam, Karl-Liebknecht-Str. 24-25, 14476 Potsdam, Germany

**Keywords:** stress concentration, on-demand release, sequential release, polymer, multifunctional

## Abstract

Mechanical stress is recognized as a principle for opening enclosed compartments through compression, stretching, or shear, eventually resulting in the onset of a diffusion-controlled release. Here, we hypothesized that the geometrical design of cavities (cut-outs) introduced as containers in elastic polymer substrates and sealed with a brittle coating layer would enable a pre-defined release of different compounds by stress concentration phenomena. Design criteria such as cut-out shapes, orientations, and depths were initially assessed for suitably different stress concentrations in computational models. In substrates fabricated from polydimethylsiloxane by photolithographic techniques, the local strains at horizontal rectangular, circular, and vertical rhombus-shaped cut-outs systematically increased under horizontal stretching as proposed. When filled with model compounds and coated with poly(*n*-butyl cyanoacrylate), a pre-defined induced breakage of the coating and compound release was confirmed upon continuous uniaxial stretching. This proof of concept demonstrates how device design and functions interlink and may motivate further exploration in technology and medicine for deformation-induced on-demand dosage applications.

## 1. Introduction

It is key for pharmaceutical products to provide active ingredients in such a manner that therapeutic efficacy is achieved. However, the timing of administration is typically put in the hand of patients for conventional products, i.e., patients are responsible for the administration schedule. Low adherence to such schedules or other types of misuse can be a cause of drug therapeutic failure [1,2]. Furthermore, there are cases where drug dosing is not required according to a pre-defined fixed schedule but should be based on the occurrence of symptoms or changes in tissue functions. In all of these cases, it would be beneficial if drug dosage occurs on-demand, i.e., in an automated manner and ideally only locally at the respective site of the body [3]. Constructing carrier systems, in particular nanocarriers, based on stimuli-sensitive materials has been one direction of research to realize on-demand drug delivery [4]. Furthermore, stimuli-responsive macroscopic implants have been evaluated [5], for which the elution of multiple drugs is an ongoing topic [6]. Combining (i) the capability of macroscopic drug dosage systems to host different substances and (ii) the ability to release drugs in response to external triggers could provide interesting on-demand release technologies. Local stress and strains, e.g., at the body surface, are examples of physical factors with high relevance for sensing [7]. In this concept study, we investigated how such mechanical triggers, in particular stress concentration, could be useful for multi-component on-demand dosage systems.

In many cases, the drug-release function of pharmaceutical implants or patches is based on passive diffusion [8], which is the underlying principle also for many on-demand release systems [9]. There are several examples where mechanical forces, as created by ultrasound [10] or local differences in osmotic pressures [11], have allowed for modulation of the release of a specific substance in an autonomous manner in a contact-free mode. Mechanical force-activated dosage systems can also use the direct contact/impact of a (typically external) load on the device [12,13,14]. By compression, the substance(s) enclosed in larger cavities or pore volumes can be forced out of the interior of the devices [15,16]. Stretching of devices can accelerate the diffusion of the substance of interest, such as through an altered surface-area–volume ratio [17] or the (reversible) opening of pores in coating materials [18,19]. Stretching can also break open a coating layer, thus enabling diffusion of a substance from the interior of multilayer systems, as it has been realized for hydrophilic cores covered with a spray deposited superhydrophobic coating [20]. In such systems, crack initiation and propagation randomly distribute over the device surface, opening diffusion pathways for any incorporated compound. Importantly, those systems did not provide a possibility to *independently* tailor the release of different substances. Therefore, it is an important challenge to establish concepts by which the on-demand release of different substances from the same device may be realized in a predefined order.

Microstructured dosing systems with several containers that are filled with identical or different substances might be a basis for a sequential release function. In order to be functional, a suitable pathway for selective opening of the containers would need to be implemented. In complex devices driven by electronic components, a burst of the coating layer for compound release could be induced by an internal pressure build-up [21]. Other electronically driven microdevices have used a melting of metal-based sealing layers to induce release [22]. Here, we hypothesized that a sequential release of compounds upon unidirectional deformation may be realized from polymer-only devices by aid of stress-concentration principles.

Stress concentration in materials is a common issue in various load-bearing structures as it is linked to a potential mechanical failure. Thus, unnecessary cut-outs or contaminating inclusions are typically to be avoided, as externally applied forces will (re)distribute around the obstacles, reaching potentially critically high stress values at their edges [23]. For instance, formerly square-shaped airplane windows, which have caused fatal accidents by crack initiation at the corners, crack propagation, and eventually fatigue of the fuselage [24], are now generally of oval shape. In some cases, however, certain geometries acting as stress concentrators are built in devices by purpose, e.g., v-shaped cuts in tear-open packages, or are researched to create aligned surface patterns [25], to grow nanowires [26], or to fabricate master molds for nanofluidics [27]. These examples indicate that stress concentration, if implemented in the design of devices, may allow for realizing distinct device functions.

Based on our hypothesis, cavities introduced into a polymeric substrate such as an elastic polymer film can be of different geometrical shapes, depths, and orientations relative to an anticipated stretching direction. Therefore, if properly selected and combined, these cut-outs can be assumed to show different extents of stress concentration, eventually resulting in different local strains upon exposure to an external stretching force. The cut-outs can also serve as containers for substances, with different substances being loaded in cut-outs of different geometries in particular, and may be sealed by a brittle coating material. In this way, according to our concept, a stretching deformation applied to the elastic polymer substrate should allow a geometry-dependent consecutive opening of sealed containers for an on-demand release of different substances from the same device.

**Figure 1:**
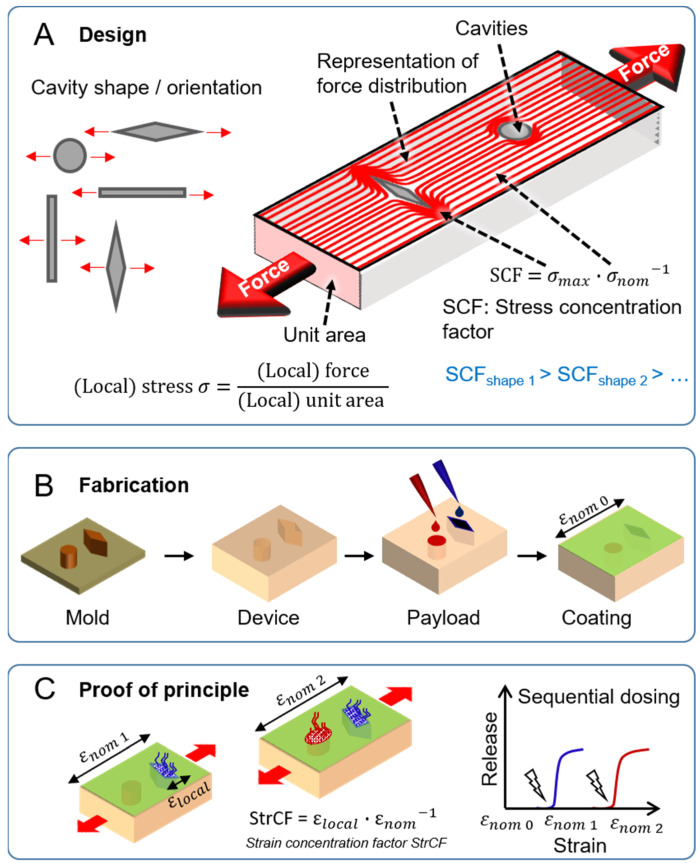
Concept and strategy for stress-induced sequential release systems based on the shapes of cut-outs (cavities) as geometrical cues. (**A**) Design of devices with cavities of distinct shapes and orientation (relative to externally applied force indicated by red arrows), affecting force distribution (represented as red lines) and causing stress concentration at specific sites of the samples (*σ_max_*: maximum stress at specific locations; σ_𝑛𝑜𝑚_: nominal stress at the unstructured cross-section area). (**B**) Device fabrication using a mold prepared by photolithography, followed by cavity loading and coating. (**C**) On-demand consecutive release of payload via strain concentration.

The strategy to evaluate the hypothesis and identify structure–function relationships involved three steps (Figure 1): First, in the design phase, materials were selected and, aided by the investigation of cut-out shapes on stress concentration in Finite element analysis (FEA), device designs with increasing numbers of cut-outs of different values of stress concentration were proposed. Second, via photolithography, devices were prepared, and their compound loading and coating were investigated. Finally, the device function for stretching-induced sequential release needed to be demonstrated.

## 2. Methods and Materials

### 2.1. Finite Element Analysis

Finite element analysis (FEA) was performed with Autodesk Inventor (version 2018.2) to quantitatively determine the distribution of von Mises stress in microstructured polymer substrates and coatings. Stretching with an uniaxial force of 0.1 N was computationally performed in the linear elastic range by applying the force to the right side of the sample, while the left side was constrained. For analysis of coated samples, the coating layer was computationally isolated from the substrate for determining von Mises stress at the coating interface. Input and output parameters are summarized in Supplementary Table S1.

### 2.2. Device Preparation

For masks, silicon wafers were spin-coated with 3 mL of SU-8 2150 photoresist (Micro resist technology GmbH, Berlin, Germany) at 500 rpm for 10 s with an acceleration of 100 rpm/s, followed by 1000 rpm for 30 s with an acceleration of 300 rpm/s. The coated wafers were heated on a programmable hotplate (HP 30 A, BlackHole Lab, Paris, France) at 65 °C for 15 min, followed by 100 °C (ramping rate ≈ 1.5 °C/min) for 4 h. Photomasks designed by Autodesk software were printed at 128k dpi (JD Photo Data company, Hitchin, UK), placed on the coated wafer, and exposed for 45 s to UV-light irradiation (mercury arc lamp 365 nm, intensity 114 mW/cm^2^; Solar simulator system, Abet technology) using a UV filter (cut-on 365 ± 7 nm, Laser Components GmbH, Olching, Germany). After exposure, the silicon wafers were heated at 65 °C for 10 min and then at 100 °C for 50 min. Thereafter, the structures were developed by immersing the silicon wafers in the developer (mr-Dev 600) for 15 min under shaking at 50 rpm/min. Then, the silicon wafers were rinsed with isopropanol and dried via an air stream.

### 2.3. Device Replication

Polydimethylsiloxane (PDMS) base and curing agent (Sylgard 184) were mixed (10:1, *w*/*w*), poured onto the masks, degassed under vacuum for 10 min, and cured in an oven at 100 °C for 35 min. The PDMS devices were peeled from the masks. The dimensions of the substrates were characterized by optical microscopy. The depth of the cavities was determined by scanning electron microscopy (SEM) (Phenom G2 pro, Phenom-World B.V., Eindhoven, The Netherlands).

### 2.4. Device Loading and Coating

Prior to loading/coating, the PDMS substrates were exposed to dopamine hydrochloride (2 mg·mL^−1^ in 10 mM TRIS buffer pH = 8.5; r.t., 24 h, shaking 50 rpm) for formation of a thin intermediate polydopamine layer as adhesion enhancer [28] and subsequently washed with water. The microinjection system used for the loading of model compounds into the device cavities under an inverted light microscope (DMI6000 B, Leica, Wetzlar, Germany) comprised a micromanipulator (SM 3.25, Märzhäuser, Wetzlar, Germany), a micro-syringe pump controller (Micro 4), a micro-injector (ultra micro pump 3—UMP3) (World Precision Instruments GmbH, Friedberg, Germany), and a syringe (Hamilton SYR 100 μL/1710RN) connected to a capillary glass (orifice of 30 μm; 1 mm outer diameter). At injection rates of 5 nL·s^−1^, ~25 nL of a mixture (9:1, *v*/*v*) of PEG (M_w_ 200 g·mL^−1^) with either 40 mg·mL^−1^ aqueous fluorescein sodium or 30 mg·mL^−1^ aqueous methylene blue was introduced in the device cavities. Coating was performed by twice spreading 5 µL solution of 𝑛-butyl cyanoacrylate in hexane (1% *v*/*v*; Cyberbond 7000, Wunstorf, Germany) over the cavities at r.t. with subsequent solvent evaporation for 1 h under a fume hood.

### 2.5. Device Characterization

Coating thickness and morphology were studied by SEM on a Phenom G2 pro (Phenom-World B.V, Germany). Coated devices were frozen with liquid nitrogen, cut with a razor blade, immersed in water to remove the payload from the cut cavities, and placed under a fume hood overnight for water evaporation. Thereafter, samples were treated in vacuum (5 × 10^−2^ mbar) for 24 h and sputter-coated with gold (5 nm; SC7640, Quorum Technologies Ltd., Lewes, UK), which was followed by SEM examination. Image analysis was performed by ImageJ program (version 1.49, National Institutes of Health, Bethesda, MD, USA).

Tensile experiments were performed with a customized miniaturized stretching device based on a tubing clip of Hofmann pattern, having one stable and one movable arm, and a micrometer screw moving the latter. Each full rotation of the screw gave an average strain (increase distance between the arms) of 5.4 ± 0.2% (determined from n = 5 individual rotations). Devices were clamped upside-down in the device, placed in a water bath at r.t., and monitored by a high-speed microscope (Keyence VW-9000 with VW-300 C, Neu-Isenburg, Germany). The recorded video was converted to snapshot images (1 image per second) and used to determine the experimental average strain rate of 0.5 ± 0.2%·s^−1^ (n = 3), illustrate the onset of release, and characterize the release kinetics after conversion to gray images (8 bit; ImageJ).

## 3. Results and Discussion

### 3.1. Device Design and FEA Analysis

According to our hypothesis, the devices needed to be designed in such a way that different levels of local stress concentration occur depending on the respective shapes of cavities in a substrate material. In the first subsection of this work, a substrate material was selected and computational tools were employed to identify the arrangements of cut-out structures that were expected to show well-separated strain concentrations for different cavities. This procedure was expected to speed up and efficiently structure the device design compared to experimental trial-and-error approaches.

**Figure 2:**
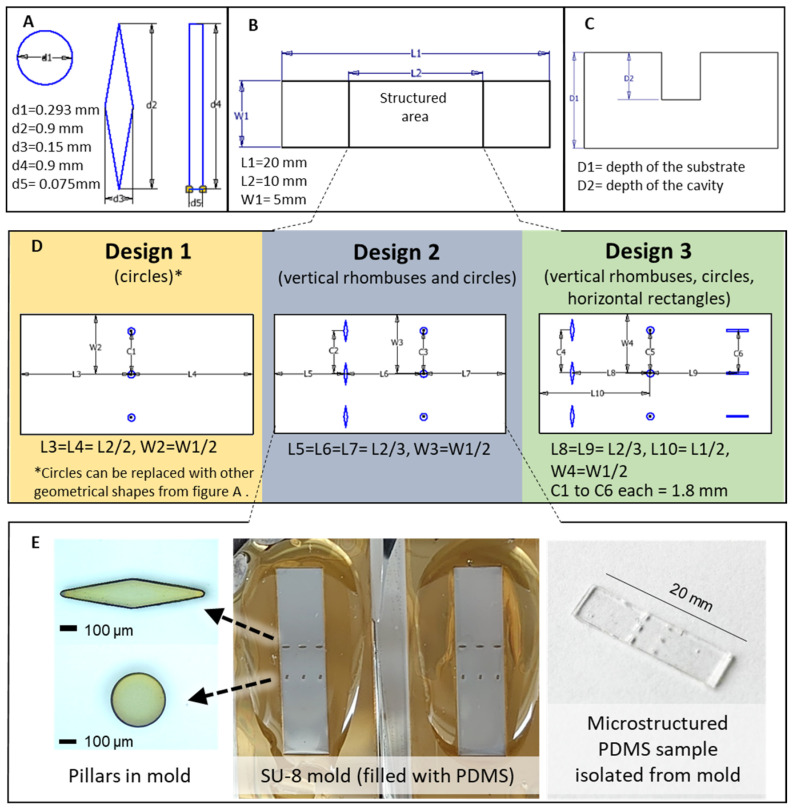
Design parameters for devices comprising (**A**) the geometrical shapes and dimensions of intended cavities, (**B**) the device dimensions, (**C**) cavity depth relative to substrate depth, and (**D**) orientation and number of cut-outs, amounting to one type in design 1 (horizontal or vertical rhombuses, horizontal or vertical rectangles, or circles), two types in design 2 (fixed combination of vertical rhombuses and circular cavities), and three types in design 3 (fixed combination of vertical rhombuses, circles, and horizontal rectangles). (**E**) Experimental realization: molds from SU-8 resin with pillars of different shapes prepared on silicon wafers by photolithography and filled with PDMS; a picture of microstructured PDMS substrate as isolated from the mold.

The substrate material of the device needed to be deformable, from which the requirement of an elastic polymer could be derived. Based on its reasonable mechanical properties, silicone rubber was used as a model material for FEA studies aiming to identify suitable device designs for sequential release. As not only cut-out shapes but also orientation relative to the stretching direction can affect stress concentration [29,30], isotropic and anisotropic shapes were selected for the simulation study, each having the same cross-sectional area. In addition to circles (isotropic), squares have been considered as a starting point for two additional shapes that would strongly differ based on stretching orientation (Figure 2A): When squares are stretched to high aspect ratios, the obtained rectangles were assumed to strongly differ in stress concentration when their short side (horizontal rectangle) versus their long side (vertical rectangle) was perpendicular to the stretching direction. The 45° rotation and compression of a square leads to rhombuses, which again can be studied with their long axis (horizontal rhombus) or their short axis (vertical rhombus) placed perpendicular to the direction of deformation. In the model, the selected shape was placed as a cut-out of a defined depth in the central region of the substrate (Figure 2B,C), using three identical cut-outs in a line perpendicular to the stretching direction. 

**Figure 3:**
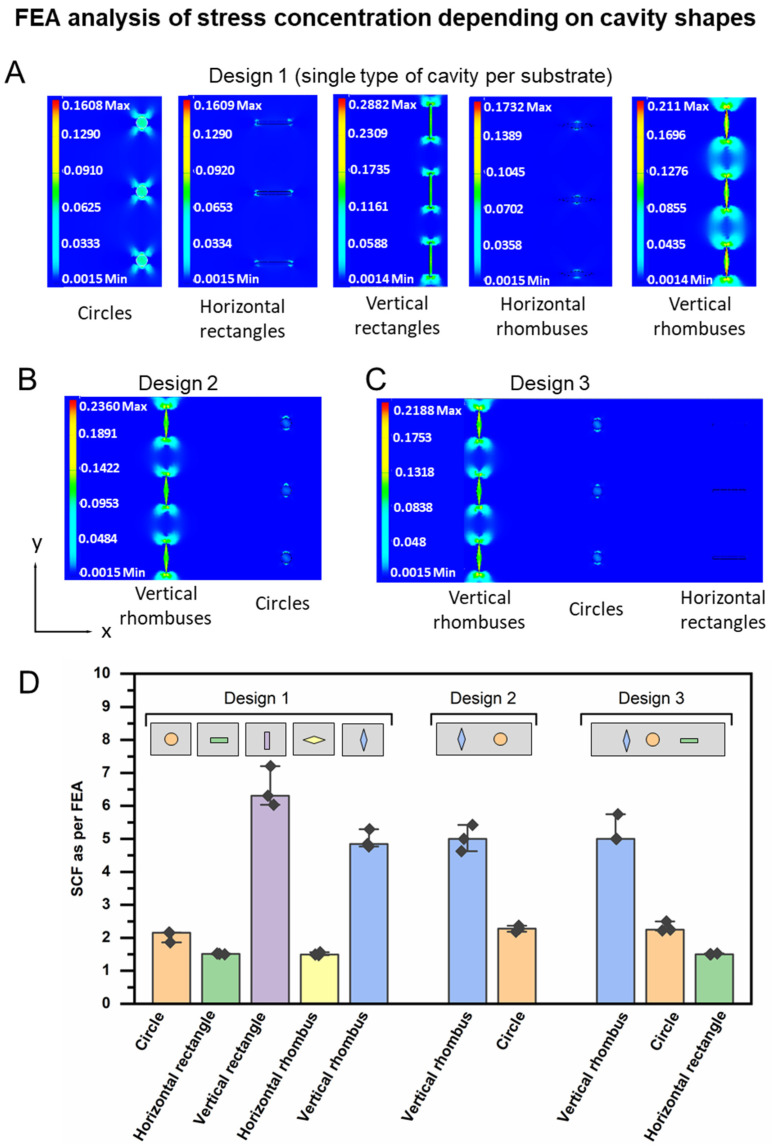
FEA analysis of von Mises stress distribution in microstructured substrates upon computational deformation with an uniaxial tensile load in the elastic range. (**A**) Devices of design 1. From left to right: cavities shaped as circles, horizontal rhombuses, vertical rhombuses, horizontal rectangles, and vertical rectangles. (**B**) Devices of design 2: vertical rhombuses cavities and circular cavities. (**C**) Devices of design 3: vertical rhombuses, circles, horizontal rectangles. The applied load was 0.1 N. The colored scale bars represent the stress intensity (unit: MPa), covering the range of stresses observed in the respective geometry (note: different scales required for panels (**A**–**C**)). For detailed parameters, see Supplementary Table S1. (**D**) Calculated *SCF* based on Equation (1) showing the median of *σ_max_*, range and the individual values for n = 3 parallel deformed cavities of a given shape according to (**A**–**C**).

In the first step, device design 1 (Figure 2D) with one type of cut-outs was investigated by FEA in the linear elastic range (for input parameters, see Supplementary Table S1), while the cavity depth was fixed in this set of computational analysis to be 90% of the substrate depth. The substrates were constrained from the left side and subjected to a tensile load of 0.1 N from the right side. The von Mises stress intensity, which corresponds to the stress distribution in 3D (multi-axial stress state), was visualized (Figure 3A). The stress concentration factor *SCF* was calculated according to Equation (1),

(1) $$\mathrm{SCF}=\frac{\sigma_{max}}{\sigma_{nom}},$$

from the maximum stress at the cutout *σ_max_* and the nominal stress at the unstructured cross-sectional area *σ_nom_*. 

Circular cavities displayed a median *SCF* of 2.1, while horizontal rectangles and rhombuses exhibited a similar median *SCF* of 1.5. A reorientation of the rectangular and rhombus cavities to a vertical direction elevated the median *SCF* to 6.3 and 4.8, respectively (Figure 3D). When combining vertical rhombuses and circles in design 2 or vertical rhombuses, circles, and horizontal rectangles in design 3, the order of stress concentration at the different cavities was not altered in the FEA model between design 2 and 3 (Figure 3B,C). The data suggest that the combinations of different shapes may not only result in different local stresses during substrate stretching but could also translate in different local strains, as should later be experimentally investigated. The observed cavity shape-dependent corona of stress concentration spreading around the cavities indicates that different maximum packing density of cavities may be possible for different shapes (e.g., reduced distances, e.g., for circles). This maximum packing density without interference patterns may be computationally optimized to increase the available cavity volume for payload.

In the next step of the FEA analysis, the impact of different cavity depths (compare Figure 2C) on the *SCF* was determined for design 2, while the substrate thickness *D_substrate_* was fixed to 0.5 mm. All cavities at a given ratio of *D_cavity_*/*D_substrate_* had the same top surface areas and volumes. Increasing *D_cavity_*/*D_substrate_* from 0.1 to 0.9 (cavity depth of 0.05 mm to 0.45 mm), i.e., from small up to very deep cut-outs, showed clear distinction of *SCF* for circular and vertical rhombus-shaped cavities (Supplementary Figure S1). *SCF* increased at both types of cavities at different slopes, thus showing the highest *SCF* differences at the maximum reasonable cavity depth of 90% relative to substrate thickness.

Eventually, according to the concept of this study (Figure 1C), the geometry-dependent build-up of stress at the site of cut-outs needed to be transferred to a rupture of the coating layer. Therefore, this coating needed to intentionally exhibit a mismatch in mechanical properties relative to the substrate, as stiff coatings on ductile substrates are known to rupture [31]. Based on the set of experimental and conceptual reasons that are elaborated in Section 3.2, poly(*n*-butyl cyanoacrylate) (PBCA) was selected for an FEA coating-layer model.

**Figure 4:**
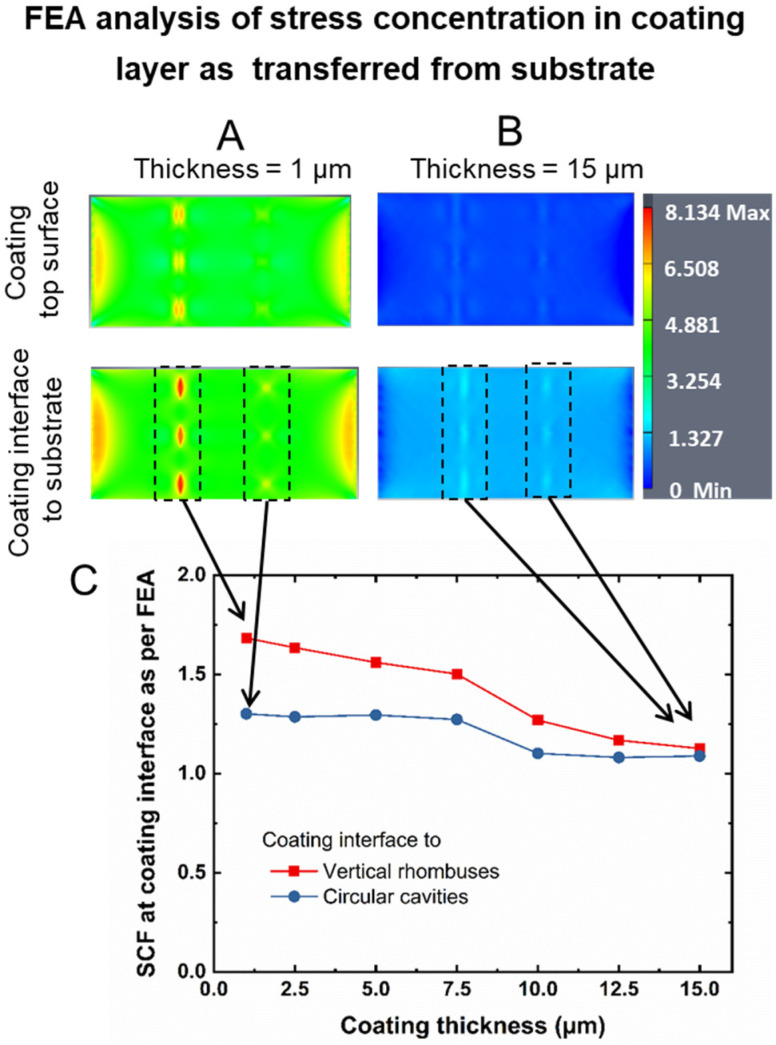
FEA of von Mises stress in coating layers above substrates of design 2 (vertical rhombuses and circles cavities) coated with PBCA. Images of stress distribution for (**A**) 1 μm and (**B**) 15 μm coating thickness. The colored scale bar represents the stress intensity (unit: MPa). Dashed squares indicate the area with local concentrated stress at interface over geometrical cavities. (**C**) Effect of coating thickness on *SCF* at coating interface at sites of geometrical cavities. The *SCF* was quantified based on the stress in the coating layer.

In this FEA model, it was assumed that the coating layer covers the structured area of the substrate (Figure 2B). The coating layers were isolated by the FEA software for visualizing and quantifying the von Mises stress at the interface. When the coating thickness was 1 μm, a locally concentrated stress appeared both at the interface between substrate and coating as well as at the surface of the coating, i.e., stress could be transferred through the coating material as desired (Figure 4A, top image). When the coating thickness was increased in this model to 15 μm, the local stress concentration appeared to be substantially reduced in the FEA visualization (Figure 4B). In a systematic computational variation of coating thickness, the *SCF* based on the von Mises stress—here, in the coating layer rather than the substrate—was assessed for device design 2 (Figure 4C). A rather similar *SCF* of ~1.1 was derived for both cavity shapes of design 2 at a coating thickness of 15 µm, which may be attributable to the stress distribution in the increased cross-sectional area of the coating. In contrast, when working with thinner coating layers, distinguishable *SCF* of ~1.7 and ~1.3 were determined at the substrate/coating interface in the position of vertical rhombuses and circles, respectively.

Thus, FEA suggests that different *SCF* depending on the geometry of cut-outs would be effective to realize a distinguishable stress concentration in a coating material, assuming that this coating is sufficiently thin. At the same time, this analysis indicates that a coating technology will be required for experimental proof-of-concept studies that allows for the realization of initially intact, thin, and brittle coatings of the cavities.

### 3.2. Device Fabrication and Coating Strategy

In this subsection of the paper, the information on the effect of cavity shapes on stress concentration as derived by FEA should be transferred to experimental verification. The goal was (i) to show that (combinations of) well-defined structures can be prepared as microstructured devices and (ii) to explore how these cavities can be sealed by a coating, for which an experimental methodology should be evaluated and coating characteristics should be tested. The procedures identified in this subsection are the basis for functional evaluation of the desired on-demand release functionality.

The experimental realization was based on the preparation of microstructured master templates from photoresist on silicon wafers and their replication by soft lithographical techniques according to design proposals from FEA. PDMS served as the matrix material for the demonstrator devices investigated in the subsequent parts of the study. This selection was based on its established use for creating microsized features with high reproducibility and its matching range of mechanical properties to the values considered in the FEA investigations [32].

The obtained PDMS devices had average dimensions of 20 ± 0.1 mm length, 5 ± 0.1 mm width, and 0.500 ± 0.09 mm thickness (n = 3) as intended (Figure 2E). The cavities, which were opened from the top and closed from the backside, were prepared with an average depth of 0.350 ± 0.05 mm (n = 3), i.e., a ratio of 𝐷_𝑐𝑎𝑣𝑖𝑡𝑦_/𝐷_𝑠𝑢𝑏𝑠𝑡𝑟𝑎𝑡𝑒_ = 0.7, selected to correspond to a high *SCF* (Supplementary Figure S1). The replication precision of the different shapes was considered acceptable; e.g., for design 2, individual circular cavities exhibited a microscopically determined surface area of ≈ 8.5∙10^−3^ ± 0.055∙10^−3^ mm^2^ (n = 3), and individual vertical rhombuses had a surface area of ≈ 9∙10^−3^ ± 0.2∙10^−3^ mm^2^ (n = 3).

**Figure 5:**
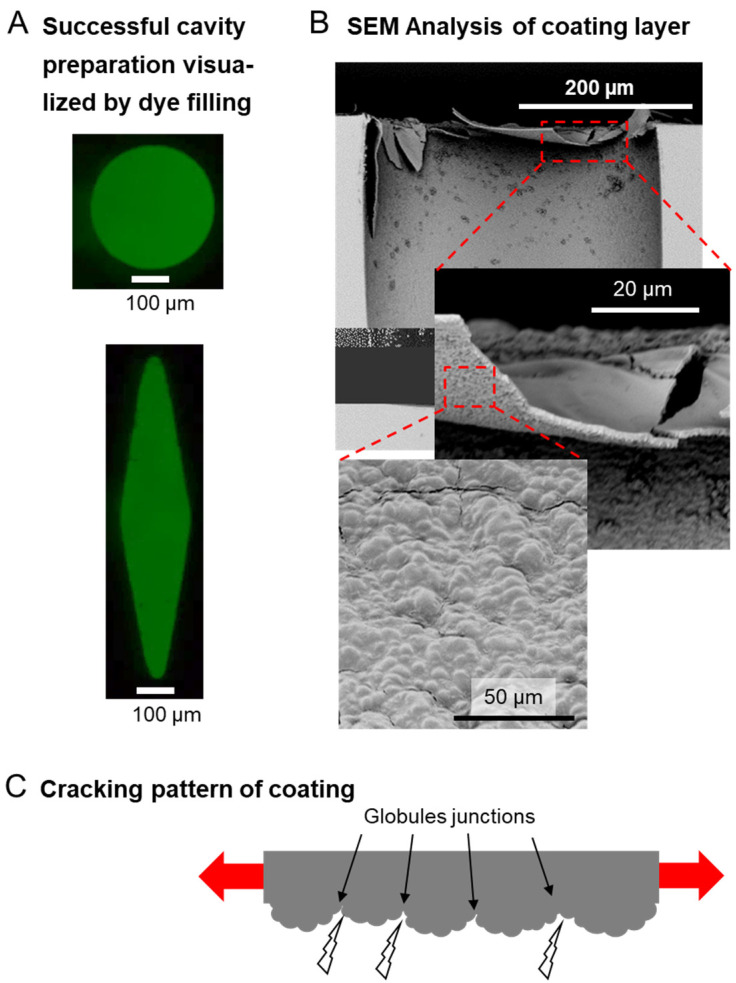
Loading and coating of devices. (**A**) Top view on cavities exemplarily filled with fluorescein dye by microinjection. (**B**) SEM analysis of a cross-section of a coated circular cavity. The sample was stretched before preparation for SEM analysis. (**C**) Scheme of inhomogeneous, globular PBCA coating structure at its bottom surface that may support crack initiation and propagation.

One key aspect to realizing the sequential release function is the practical realization of a coating layer that fulfills the requirements of a thickness of only a few micrometers (compare Figure 4), initial non-permeability, and, by its structure, support for crack initiation/propagation. As the coating cannot be applied over empty cavities, the introduction of the payloads needed to be integrated into the overall coating concept. Hence, each cavity was carefully filled using dissolved dyes mixed with poly(ethylene glycol) (PEG) via microinjection under microscopic control. The selection of PEG as filler was based on (i) the possibility to tailor the viscosity of the payload mixture depending on PEG molecular weight and (ii) good aqueous solubility, while other types of fillers may be selected as long as they do not hinder cavity deformation by local strain. Successful cavity filling via the strategy employed here was confirmed by optical microscopy (Figure 5A).

While many techniques have been established to prepare thin polymer coatings in principle, such as spin-coating, slot-die coating, or vapor deposition [33,34,35], these techniques did not appear to be suitable for the prefilled demonstrators explored here for several reasons, e.g., the payload potentially being lost during agitation. Most of these techniques typically result in relatively homogeneously structured coating layers, which would not necessarily be advantageous for initiating the rupture of the brittle (low elongation at break *ε_b_*) and relatively hard (high Young’s modulus) polymeric coating material. Furthermore, preformed polymers typically require certain types of solvents for deposition, which might swell PDMS, partially mix with the payload solution, or cause other types of interference that are difficult to control. Therefore, the selected PBCA appeared to be a particularly suitable coating option for proof-of-concept studies: as cyanoacrylates can polymerize in the presence of nucleophiles groups, PBCA is formed in situ at the substrate surface. Based on this polymerization initiation at the interface, an internal granular structure of the coating can be obtained [36], where the junctions between granules may act as notches for crack initiation and propagation. Considering the high glass-transition temperature of PBCA (118 °C [36]), the coating material will also be in its glassy (brittle) state during device usage at ambient conditions. 

The coating procedure was conducted after pretreatment with polydopamine by spreading water-immiscible hexane solutions of *n*-butyl cyanoacrylate on the substrates with filled cavities, which resulted in polymerization and deposition of hexane-insoluble PBCA. SEM indicate the presence of a smooth top surface of the PBCA film without pores. SEM analysis estimated the coating thickness to be 2 ± 0.9 µm (n = 6), which is in the preferred range as derived from FEA analysis. Additionally, the proposed globular structure of the side of the PBCA film facing the substrate was confirmed (Figure 5B,C).

### 3.3. Functional Evaluation of Sequential Release Capability

The functional evaluation of the prepared devices was the goal of this subsection; specifically, the extent and order of local deformations at the site of cavities needed to be examined under external mechanical load to verify the FEA-based design hypotheses. Subsequently, as the final part of this concept study, the capability of the proposed technology for on-demand sequential release of different substances from the same device in a predefined order was tested. This dataset was expected to facilitate a conclusion on the general suitability of stress concentrations in microstructured devices for multi-dose or multi-drug release by sensing and responding to mechanical triggers.

**Figure 6:**
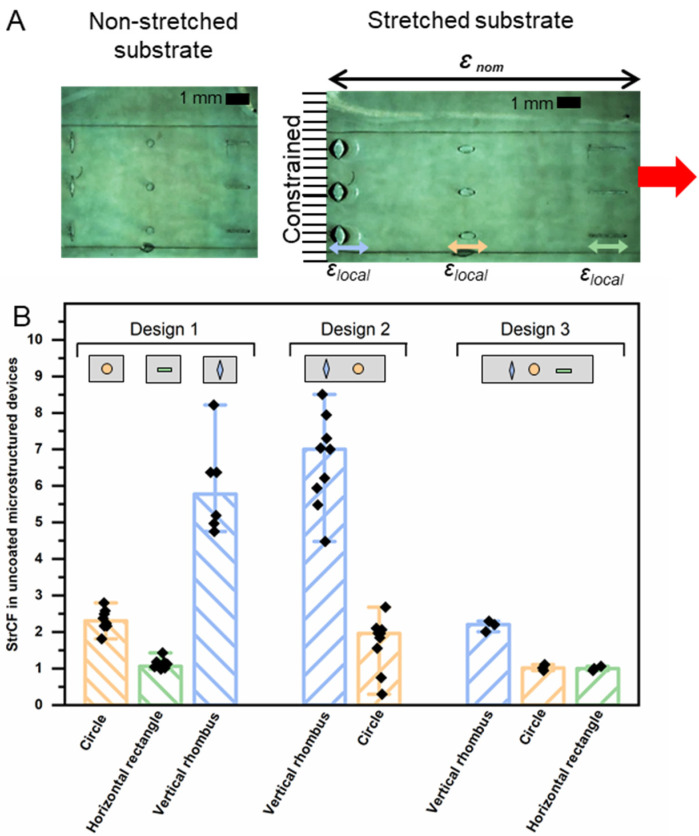
Experimental analysis of strain concentration at cavities depending on the geometrical design. (**A**) Exemplary images of non-stretched and stretched (*ε_nom_* = 60%) substrates of design 3 comprising, from left to right, vertical rhombuses, circles, and horizontal rectangles. Local strains are indicated by the colored arrows and are normalized to the initial cavity dimension of the non-stretched substrate prior to the calculation of the *StrCF*. (**B**) Experimental *StrCF* calculated according to Equation (2) based on the analysis of microscopic images. Data represented as median and range (n = 3–9).

High local stress in the polymer substrate mediated by the specific geometries of the cavities was expected to result in a local deformation (strain) that was higher than the nominal strain of the substrate as described by the strain concentration factor *StrCF*, as described by Equation (2):

(2) $$StrCF=\frac{\varepsilon_{local}}{\varepsilon_{nom}},$$

where *ε_local_* represents the strain observed at the side of the respective cut-out, and *ε_nom_* is the nominal overall strain of the substrate. In the first step of the experimental evaluation, non-coated samples of designs 1, 2, and 3, where the local deformation could be microscopically visualized due to the absence of the coating materials, were assessed at *ε_nom_* = 60% for their *StrCF* (Figure 6). In case of design 1 with single shapes only, i.e., either horizontal rectangles or circles or vertical rhombuses, the latter showed the highest experimental *StrCF*. For design 2, with circles and vertical rhombuses in the same device, the strain was highly concentrated at rhombus-shaped cavities as expected (median *StrCF* = 7.3; range 1.5), while it was reduced at the circular cavities (median *StrCF* = 2.1; range 0.7) compared to the data obtained for design 1. Combining all three types of cavities together in design 3 resulted in lower *StrCF* for all geometries, which may be attributed to a distribution of the applied strain among the cavities that reduced the concentrated strain for each of them. Importantly, the *StrCF* of vertical rhombuses continues to stand out as the shape of the highest magnitude of *StrCF* (median *StrCF* = 2.2), followed by similar values for circles and horizontal rectangles (median *StrCF* = 1.0). Thus, there may be boundaries when combining more than two shapes in close proximity to each other in strain-sensing release systems. 

Overall, this analysis with bare microstructured substrates experimentally proved the proposed mechanism in that combinations of geometrical cues cause differences in local deformation in soft materials, which has predominantly been investigated for single holes in metals or polymers in the past [37]. As part of future studies, the contribution of the globular PBCA film structure and potential residual stress originating from the in situ polymerization on the coating breakage could be investigated, e.g., via tensile tests in SEM. However, such investigations in comparison to PBCA coating deposition by thin-film solvent casting techniques will be highly challenging considering the brittleness of PBCA and the difficulties in obtaining and handling the material as free-standing, very thin films.

**Figure 7:**
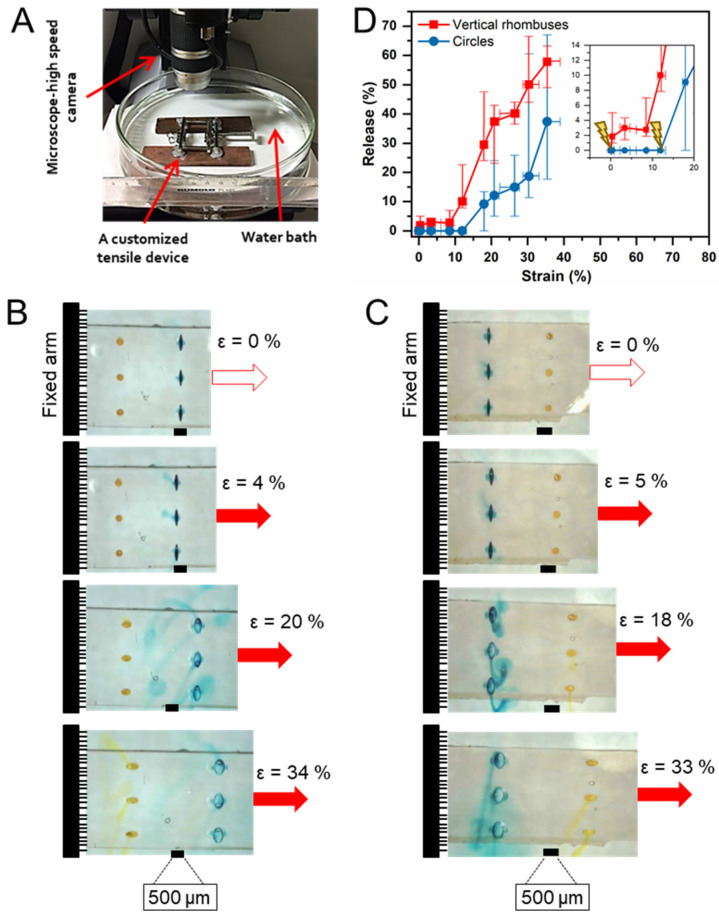
Stress-induced sequential release function from dye-loaded, PBCA-coated devices (design 2). (**A**) Set-up for stretching devices in water environment combined with high-speed microscopy imaging. (**B**) Image series from stretching experiment of devices fixed with vertical rhombus-shaped cavities close to the movable arm of the tensile instrument. (**C**) Image series from stretching experiment of devices fixed with circular cavities close to the movable arm of the tensile instrument. Scale bars = 500 µm. (**D**) Quantitative assessment of release during continuous stretching based on grayscale image analysis. Data represented as median and range (n = 3), stretching rate 0.5 ± 0.2 %/s. The error of the customized tensile device was determined to be 11%.

To demonstrate that the differences in strain concentration enable a sequential release functionality, two dyes were loaded in devices of design 2 using fluorescein sodium in circular cavities and methylene blue in vertical rhombuses. The coated devices were clamped into a customized manual tensile device in a water bath at ambient conditions and subjected to a slow and continuous stretching deformation of about 0.5 ± 0.2%·s^−1^ while being monitored with a high-speed camera system (Figure 7A). Upon continuous stretching, the rhombus-shaped cavities opened first as hypothesized, followed by the circular cavities (Figure 7B). To demonstrate that the geometrical cues and not the location of the cavity relative to the fixed or movable arm of the tensile instrument affect the sequence of release, a second set of experiments was conducted with inversed sample orientation. This analysis resulted again in the release initiation from rhombus-shaped cavities prior to circular cavities (Figure 7C), thus confirming the underlying principle of stress and strain concentration.

Grayscale image analysis [38] has been conducted to quantify the release in correlation to the applied strain (for methodology, see Supplementary Figure S2) for three cases of devices: (i) design 1 with circular reservoirs only, (ii) design 1 with vertical rhombuses as reservoirs only, and (iii) design 2 containing circles and vertical rhombuses. The analysis illustrated that the release was initiated for design 1 at 𝜀 ≈ 0.6% (median) for rhombus-shaped cavities and at 𝜀 ≈ 5% (median) for circular cut-outs (Supplementary Figure S3). Upon combination of these cavities in design 2 devices, the release onset from the rhombus reservoirs was again noted at low ε ≈0.4% (median), while the release from circular reservoirs started at 𝜀 ≈ 12 (median). This shift of release-initiation of circular cavities from 𝜀 ≈ 5% (design 1) to 𝜀 ≈ 12% (design 2) is an expected consequence of the strain concentration in design 2 first affecting the vertical rhombuses according to their higher *SCF*, thus matching predictions by FEA (Figure 3D) and the investigation on neat substrates (Figure 6B). It should be noted that further stretching beyond the point of crack initiation contributed to the subsequent release in the selected experimental procedure of continuous stretching at a constant rate. This contribution can be justified by crack propagation and, in case of the liquid-filled and thus compressible cavities used here, by a certain reduction of cavity volume associated with substrate deformation. As crack induction due to stress concentration is the key factor for destroying the diffusion barrier function of the coating, pausing stretching after opening the first sets of cavities could be, in principle, a path to completely separating the release periods from differently shaped cavities.

## 4. Conclusions

In this study, spatially directed stress and strain control were the starting point for elaborating the *SCF*, geometry, and depth of cut-outs as design principles to enable a controlled release function of stretchable microstructured devices that can be initiated on-demand and be sequentially controlled, all being based on polymer-only devices. Cavity shapes as geometric cues, which direct the order of the release relative to the stretching direction, may open up a rich design space that can be elaborated on in the future. Applications may range from the dosage of compounds such as pharmaceutics (e.g., to the skin) or reagents like catalysts or glues for self-repair to strain sensors visualizing critical overstretching, e.g., of artificial skin in soft robots. In addition to the release of a larger number of different compounds, a stretching-direction-dependent release sequence could also be a subject for ongoing research that may benefit from the model-driven systematic device design presented here.

## Data Availability

The raw/processed data required to reproduce these findings are available from the corresponding authors upon reasonable request.

## Supplementary Materials

The following supporting information can be downloaded at https://www.mdpi.com/article/10.3390/pharmaceutics14122611/s1: Figure S1: FEA analysis of the effect of cavity depth on *SCF*; Figure S2: Methodology for semi-quantitative evaluation of release from images through grayscale analysis; Figure S3: Release profiles of stress-induced release from devices with single-shaped cavities (design 1); Table S1: Input parameters of FEA simulation and determined values [39,40,41].
