# Supplementary material for: Design of Reservoirs Enabling Stress-Induced Sequential Release Systems
Source: Pharmaceutics. 2022 Nov 26;14(12):2611. doi: 10.3390/pharmaceutics14122611 (PMC9781554; doi:10.3390/pharmaceutics14122611)
Supplement: Supplementary file 1 [file pharmaceutics-14-02611-s001.zip › pharmaceutics-2014396-supplementary.pdf]

Supplementary material

Supp. Table S1. Input parameters of FEA simulation and determined values

Input parameters in the simulation software	General objective and settings		Study type	Static analysis
			Modeling	Linear elastic range
			Design objective	Single point
	Substrate	Assigned material of the substrates	Name	Silicone rubber [a]
			Mechanical properties	Yield Strength 10.34 MPa
				Tensile Strength 6.5 MPa
				Young's Modulus 3 MPa
				Poisson's Ratio 0.49
				Shear Modulus 1.007 MPa
		Mesh settings	Average element size (fraction of model diameter)	0.1 mm
			Minimum element size (fraction of average size)	0.2 mm
			Local element size of all surfaces of substrates and cavities	0.1 mm
			Grading factor	1.5
			Maximum turn angle	60 degree
			Create curved mesh elements	Yes
		Operating conditions	Constraint type	Fixed Constraint at left side of the substrate
			Load type	Force
			Magnitude	0.1 N from the right side of the substrate
	Coating layer	Assigned material of the coating	Name	Poly(***n***-butyl cyanoacrylate) [b]
			Mechanical properties	Yield Strength 0.79 MPa
				Tensile Strength 0.9 MPa
				Young's Modulus 8000 MPa
				Poisson's Ratio 0.4
				Shear Modulus 0.125 MPa
		Coating type	Thin layer (shell)	
		Mesh setting	Average element size in shell is 0.05 mm	
		Operation condition	Contact type of the coating layer (shell) on the substrate is bonded	
Output of the study		Stress distribution visualization and quantification	Nominal of von Mises stress in the substrate was quantified	
			Maximum of von Mises stress at site of cavities was quantified	
			Nominal of von Mises stress in the coating layer at the coating-substrate interface was quantified	
			Maximum of von Mises stress in the coating layer over the site of cavities at the coating-substrate interface was quantified	
Calculated values		From the output of the study, *SCF* of different cavities and at coating-substrate interface was calculated by equation 1 of the main manuscript		

[a] Material properties according to Autodesk Inventor data base (version 2018.2). [b] Material properties: (i) yield strength, tensile strength, shear modulus are estimates based on Technical Data Sheet of Cyberbond CB 7000 and J. Dental Res. 48, 536-542, 1969; (ii) Poisson ratio adapted according to values noted for polyethylcyanoacrylate in J. Strain Anal. Eng Design 38: 233-245, 2003; (iii) Young's modulus according to microindentation measurements published in Polymers 14: 4863, 2022.

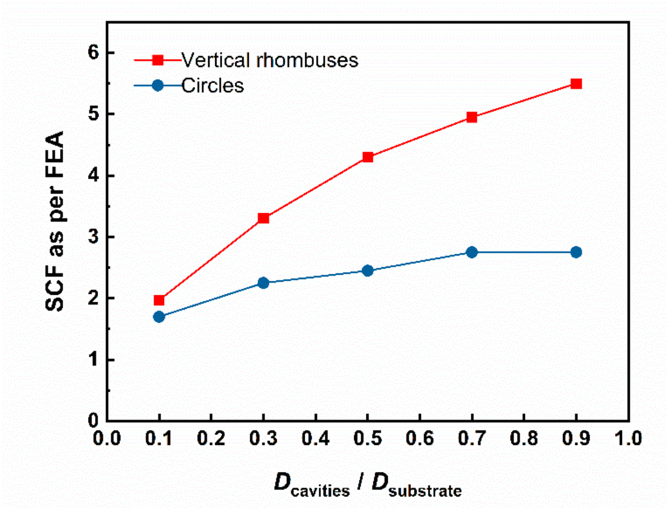

Figure S1. Result of FEA analysis. Effect of cavities depth D_{cavity} relative to the substrate thickness $D_{substrate}$ on the *SCF* as analyzed by FEA. $D_{substrate}$ was kept constant at 0.5 mm, while D_{cavity} was varied from 0.05 mm to 0.45 mm. Each substrate was subjected to a computational uniaxial load = 0.1 N in the elastic range, and the *SCF* was calculated based on mean values of the detected σ_{max} (von Mises stress) of the n=3 parallel deformed cavities. Detailed parameters see Supp. Table S1.

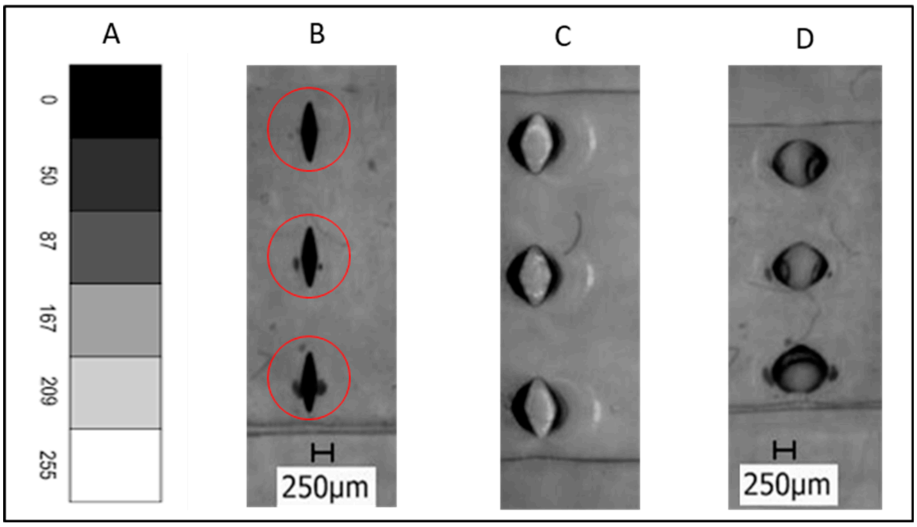

Figure S2. Methodology for semi-quantitative evaluation of release from images through grayscale analysis. (A) An image of the universal grayscale. Black = 0 gray value. White = 255 gray value. B) Initial gray value in cavities (remarked with red circles), i.e., loaded cavities before stretching (0% release), e.g., gray value = 50. C) A control gray value of cavities (unloaded cavities = 100% release), e.g. = 209 gray value. D) A gray value of cavities during tensile-release experiment, e.g., at 3% strain cavities has a gray value = 167 averaged over the area of the cavity.

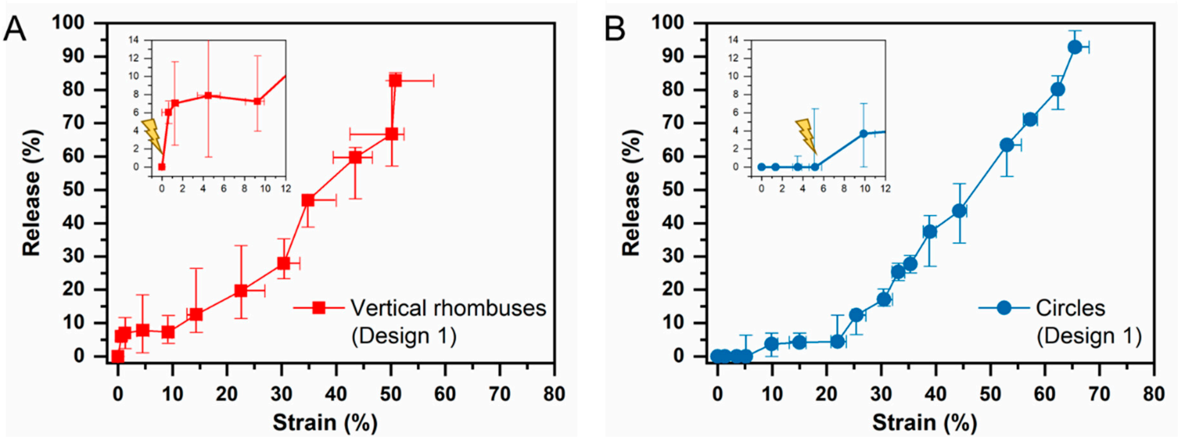

Figure S3. Release profiles of stress-induced release from devices with single-shaped cavities (design 1). (A) Release from substrates containing only rhombus cavities. (B) Release from substrates containing only circular cavities. Data represented as median and range (n = 3). The error of the customized tensile device was determined to be 11%.
